# Proton pump inhibitor has no effect in the prevention of post-endoscopic sphincterotomy delayed bleeding: a prospective randomized controlled trial

**DOI:** 10.3389/fmed.2023.1179512

**Published:** 2023-06-02

**Authors:** Zhengping Yu, Jinli He, Ronglai Cao, Zhenzhen Yang, Baolian Li, Junbo Hong, Youxiang Chen, Liang Zhu

**Affiliations:** Department of Gastroenterology, The First Affiliated Hospital of Nanchang University, Jiangxi Clinical Research Center for Gastroenterology, Nanchang, China

**Keywords:** proton pump inhibitor, endoscopic retrograde cholangiopancreatography, endoscopic papillary sphincterotomy, delayed bleeding, randomized controlled trials

## Abstract

**Background and aims:**

Bleeding is one of the common adverse events of endoscopic retrograde cholangiopancreatography (ERCP), which is mainly caused by endoscopic sphincterotomy (EST). At present, it remains unclear whether proton pump inhibitor (PPI) should be used to prevent post-EST bleeding. Therefore, we performed a randomized controlled trial to investigate whether PPI is effective in the prevention of post-EST delayed bleeding.

**Methods:**

Consecutive eligible patients were randomly assigned (1:1) to experimental group (PPI group) or control group (normal saline, NS group). The patients in PPI group received intravenous esomeprazole 40  mg and normal saline 100  mL every 12  h for 2  days after ERCP immediately, and followed by oral esomeprazole (Nexium) 20  mg once a day for 7  days. Correspondingly, patients in the control group received intravenous normal saline 100  mL and did not take PPIs or any acid-suppressing drugs during hospitalization and after discharge. All patients were followed up for 30  days after ERCP. The primary endpoint was the incidence and severity of post-EST delayed bleeding.

**Results:**

Between July 2020 and July 2022, 290 patients were randomly assigned to PPI group (*n* = 146) or NS group (*n* = 144). 5 patients from each group were excluded from the final analysis. There were 6 patients with post-EST delayed bleeding, with an incidence rate of 2.14%. The median time of delayed bleeding was 2.5  days after ERCP. 3 cases (2.12%, 3/141) occurred in the PPI group, with 1 case of mild and 2 cases of moderate bleeding. 3 cases (2.16%, 3/139) occurred in the NS group, with 2 cases of mild and 1 case of moderate bleeding. There was no significant difference in the incidence and the severity of post-EST delayed bleeding between the two groups (*p* = 1.000).

**Conclusion:**

Prophylactic use of PPI after EST does not reduce the incidence and severity of post-EST delayed bleeding in patients.

**Clinical Trial Registration:**

https://www.chictr.org.cn/searchproj.aspx, identifier ChiCTR2000034697.

## 1. Introduction

Since first described in 1974, endoscopic sphincterotomy (EST) has become a standard procedure of therapeutic endoscopic retrograde cholangiopancreatography (ERCP) and was widely used in the treatment of pancreaticobiliary diseases. Previous literature has pointed out that bleeding is a severe adverse event after ERCP, and usually occurs after EST (1). According to the bleeding time, post-EST bleeding can be classified as immediate bleeding or delayed bleeding. Immediate bleeding refers to the bleeding that occurs during ERCP, which is easy to find under endoscopy and can be dealt with promptly. Therefore, immediate bleeding was considered to have no clinical significance (2–4). Delayed bleeding refers to the bleeding that occurs several hours to several weeks after the ERCP, and mainly occurs 24–72 h after the procedure (5). Post-EST bleeding is primarily manifested as hematemesis, melena, or hemorrhagic fluid drainage from the nasobiliary tube, and the incidence ranged from 0.3 to 2.0% (6, 7). Minor bleeding is often self-limiting and can be stopped without special treatment, while major bleeding can be fatal and requires endoscopic hemostasis or angiographic embolization. Regarding the prevention of post-EST bleeding, current measures include avoiding unnecessary EST, replacing EST with balloon dilation, replacing pure cutting current with mixed current, and prophylactic injection of hypertonic saline-epinephrine solution to the papilla (8–10). As mentioned above, most studies on the prevention of post-EST bleeding have focused on technical improvement of the procedure, while studies related to pharmacological prevention are scarce. In addition, some methods for preventing bleeding may increase the risk of post-ERCP pancreatitis (11, 12). Until now, only one study has evaluated the role of medications in preventing post-EST bleeding, which showed that prophylactic proton pump inhibitor (PPI) did not reduce the risk of EST bleeding (13). However, their study did not differentiate immediate bleeding from delayed bleeding, and did not establish a placebo group, so further studies are needed to prove the result.

At present, the role of PPI in preventing post-EST bleeding remains unclear. Therefore, this prospective randomized controlled trial aimed to investigate the effect of PPI in the prevention of post-EST bleeding, and to determine whether patients need to use PPI to prevent post-EST bleeding.

## 2. Materials and methods

### 2.1. Patients

The patients who underwent ERCP procedure for the first time in the First Affiliated Hospital of Nanchang University between July 2020 and July 2022 were immediately recruited, and evaluated for clinical trial inclusion. Inclusion criteria: (1) patients who signed informed consent, (2) patients aged between 18 from 80 years, and (3) EST was performed during ERCP. If immediate bleeding occurs during ERCP, complete intraoperative hemostasis is performed so that there is no active bleeding after operation until enrollment. Exclusion criteria: (1) patients with severe hepatic insufficiency (Child-Pugh class B or C), (2) patients with severe renal insufficiency: endogenous creatinine clearance <50 mL/min, (3) patients with coagulation disorders: platelets <100 × 10^9/L, prothrombin time (PT) prolonged by more than 3 s, international normalized ratio (INR) >1.5, activated partial thromboplastin time (APTT) >1.5 times the upper limit of normal, (4) patients with peptic ulcer bleeding or gastrointestinal bleeding caused by the following causes: esophageal varices, reflux esophagitis, Mallory-Weiss syndrome, Dieulafoy disease, colon disease, small intestine disease or distal gastric ulcer after gastrectomy, (5) patients with acute pancreatitis before ERCP, (6) patients requiring anticoagulation and antiplatelet agents within 7 days before the study, (7) patients with active cardiovascular and cerebrovascular disease, (8) pregnant or breastfeeding women, (9) known or suspected allergy to any PPI, (10) patients enrolled in any study involving an investigational agent within 30 days prior to this study, and (11) patients using acid suppressive agents within 24 h before enrollment. Termination test criteria: (1) patients have adverse drug events and (2) patients request to withdraw from the trial. This study has been approved by the Ethics Committee of the First Affiliated Hospital of Nanchang University, and registered at the Clinical Trials Registration site in China (No. ChiCTR2000034697).

### 2.2. Study design

This study is a prospective, randomized, controlled clinical trial study, and two groups were established, namely the experimental group (PPI group) and the control group (normal saline, NS group). According to previous literatures, the incidence of post-EST bleeding is ranged from 0.3 to 2.0% (6, 7). It is assumed that the PPI group and the NS group are equivalent in preventing post-EST bleeding so that the probability of bleeding in the PPI group is no higher than 5% (95% confidence level) in the NS group with 80% power. By means of clinical trial formula with the dropout rate of 10%, the required sample size is at least 276 cases, 138 cases in each group. A sample size of 290 cases is planned to be included. Before starting the experiment, random assignment was performed using the random number method. The odd number represented the PPI group and the even number represented the NS group. Each number was placed in a sealed envelope and sequentially numbered for allocation concealment. The quality control nurse started from the first recipient and ordered them by number. The envelopes were opened sequentially and assigned to groups according to the agreed rules. The nurse did not participate in patient care and the researchers were blinded to the allocation. After ERCP, patients in the PPI group immediately received intravenous esomeprazole 40 mg dissolved in normal saline 100 mL every 12 h for 2 days, followed by oral esomeprazole (Nexium) 20 mg once a day for 7 days (regardless of whether the patient was discharged during this period). Correspondingly, patients in the NS group immediately received intravenous normal saline 100 mL every 12 h for 2 days after ERCP, all of whom did not use PPIs or any acid-suppressing drugs during hospitalization and discharge.

### 2.3. Treatment

ERCP was performed using the JF260V or TJF260V electronic duodenoscope and related accessories manufactured by OLYMPUS Company, Japan. For patients who were considered to be at high risk for PEP, we administered 100 mg rectal diclofenac suppositories immediately before the procedure. The patients were placed in the prone position during the operation, deeply anesthetized with propofol. During the procedure, oxygen inhalation and ECG monitoring were given. All the patients received EST treatment and were observed after EST to determine whether there was immediate bleeding. Those with minor bleeding that stopped spontaneously within a few minutes of observation need no special treatment. Patients with severe bleeding which could not ceased spontaneously were treated with ice saline rinsing, endoscopic spraying of 1:10000 ice norepinephrine saline, balloon compression or titanium clip closure according to the situation until there was no bleeding. All patients were operated by experienced endoscopists and were given routine post-ERCP care such as fasting for 24 h, rehydration, electrolytes and energy supplement, monitoring vital signs, etc. 3 h after ERCP, blood amylase was test and 24 h after ERCP, blood routine, liver function, blood amylase, coagulation function and other laboratory tests were reviewed. Symptoms such as abdominal pain, fever, hematemesis, melena, etc. were observed. If relevant symptoms appeared, whether it was an adverse event after ERCP was determined, and the patient was treated accordingly. If post-EST delayed bleeding was suspected, secondary endoscopic examination was performed to confirm the diagnosis, and appropriate hemostatic treatment such as fluid resuscitation, endoscopic hemostasis or blood transfusion was given according to the bleeding situation. All patients were followed up by outpatient service or telephone call at 1 week, 2 weeks and 1 month after discharge to ask whether they had any manifestation of gastrointestinal bleeding, and if delayed bleeding was suspected in discharged patients, they were re-admitted to the hospital for endoscopic examination to confirm the diagnosis.

### 2.4. Definition

(1) Post-EST bleeding: Patients with any one of ABC plus D can be diagnosed. A: Hematemesis or blood from nasobiliary tube drainage, or bloody stool or melena after normal stool; B: hemoglobin decreased >20 g/L within 24 h (or hematocrit >6%), or blood transfusion ≥2 units within 24 h, but hemoglobin increased <10 g/L (or hematocrit <3%); C: circulatory instability, systolic blood pressure ≤ 90 mmHg or pulse ≥110 beats per minute (reappears after circulatory stability); D: Endoscopy is clearly defined as bleeding after EST (Table 1), (2) Classification of bleeding severity: mild bleeding: clinical manifestations of bleeding, hemoglobin drop <30 g/L and no indication for transfusion; moderate bleeding: transfusion volume ≤ 4 units, no need for interventional therapy (angiographic or surgical); severe bleeding: transfusion volume ≥ 5 units, or interventional therapy (angiographic or surgical) is required (14), (3) Post-ERCP pancreatitis (PEP): new or aggravated abdominal pain with blood amylase or lipase higher than 3 times the upper limit of normal at 24 h after ERCP (5), (4) Biliary tract infection: new onset temperature > 38°C for more than 24 h combined with cholestasis or right upper quadrant signs of inflammation with imaging findings characteristic of acute cholecystitis, without any suggestive clinical or imaging findings before ERCP (5), and (5) Perforation: evidence of air or luminal contents outside of the gastrointestinal tract as determined by imaging. Types of perforation: type I: duodenal wall perforation; type II: periampullary perforation; type III: biliary or pancreatic duct perforation; type IV: retroperitoneal gas alone (5).

**Table 1:** Diagnostic criteria and severity grading of post-EST bleeding.

Diagnostic criteria	Severity grading		
	Mild	Moderate	Severe
Patients with any one of ABC plus D A: Hematemesis or blood from nasobiliary tube drainage, or bloody stool or melena after normal stool; B: hemoglobin decreased >20 g/L within 24 h (or hematocrit >6%), or blood transfusion ≥2 units within 24 h, but hemoglobin increased <10 g/L (or hematocrit <3%); C: circulatory instability, systolic blood pressure ≤ 90 mmHg or pulse ≥110 beats per minute (reappears after circulatory stability); D: Endoscopy is clearly defined as bleeding after EST	clinical manifestations of bleeding, hemoglobin drop<30 g/L and no indication for transfusion	transfusion volume ≤ 4 units; no need for interventional therapy (angiographic or surgical)	transfusion volume ≥ 5 units, or interventional therapy (angiographic or surgical) is required

### 2.5. Outcome

The primary endpoint is the incidence of post-EST delayed bleeding within 30 days. The secondary endpoints include (1) severity of post-EST delayed bleeding within 30 days, (2) percentage of patients requiring interventional or surgical treatment within 30 days, and (3) percentage of patients requiring blood transfusion within 30 days. Other indicators are adverse events except bleeding, mainly including PEP, biliary tract infection, perforation and death.

### 2.6. Data collected

(1) Patients’ basic conditions, past history, personal history, clinical symptoms and preoperative diagnosis, (2) laboratory test results before and after ERCP, (3) endoscopic findings and endoscopic procedures performed during ERCP, (4) the incidence and severity of post-EST delayed bleeding, and (5) other adverse events after ERCP.

### 2.7. Statistical analysis

The statistical analysis was performed using SPSS 25.0 software. Categorical variables data were presented as percentages (%), and the Chi-square test or Fisher exact test was used for analysis. Continuous variables data were expressed as mean ± standard deviation or median (25th–75th percentiles) where appropriate, and compared by student *t* test or nonparametric test. *p* < 0.05 was considered statistically significant.

## 3. Results

### 3.1. Participants and baseline characteristics

**Figure 1:**
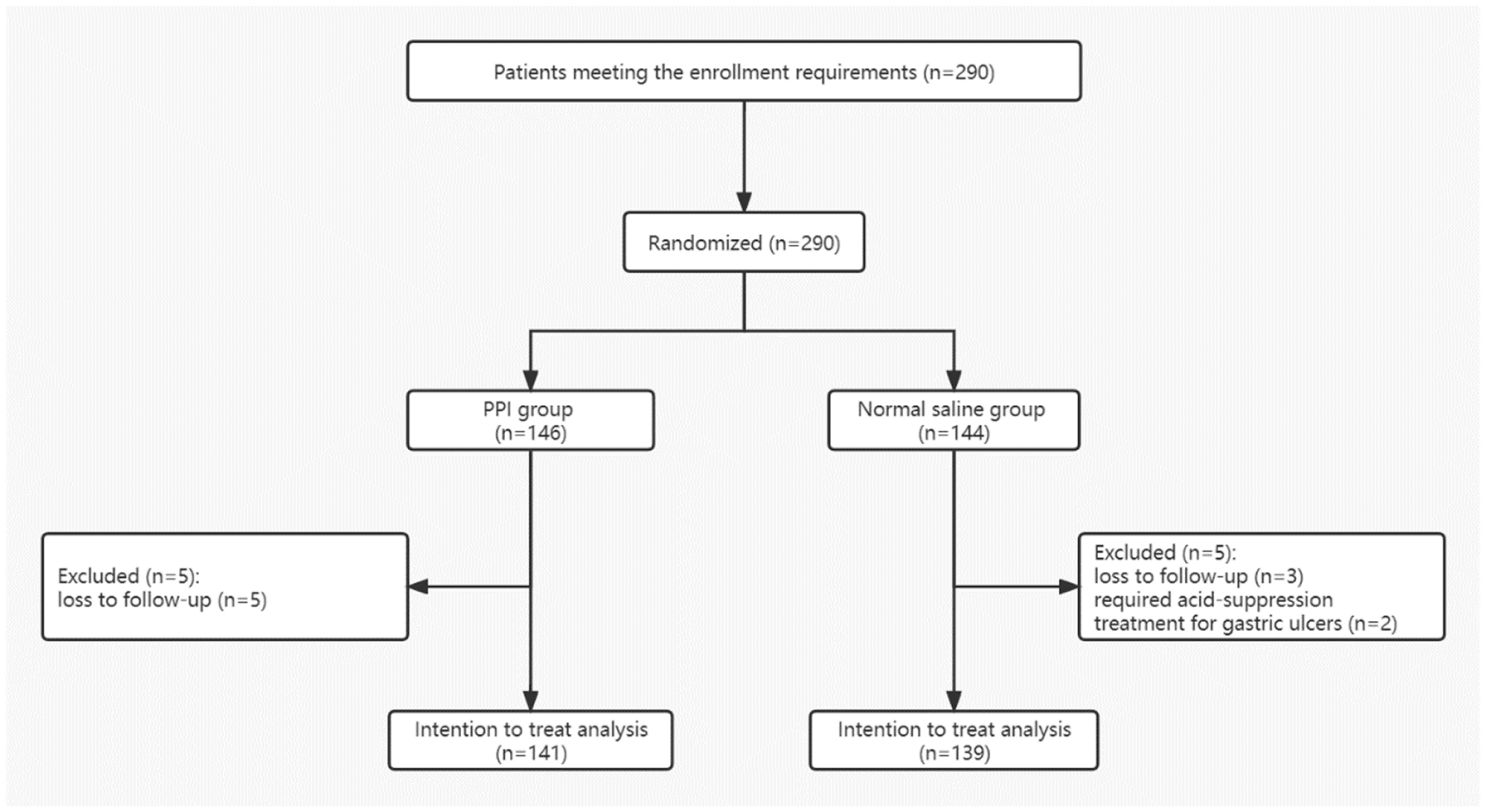
Trial flow diagram.

A total of 290 patients who met the criteria were randomly assigned according to the random number method. 146 patients were assigned to PPI group, of which 5 patients were excluded due to loss of follow-up, and finally a total of 141 patients in PPI group were included in the analysis. 144 patients were assigned to NS group, of which 2 patients were diagnosed with gastric ulcers during ERCP and required acid-suppressive medication so they were withdrawn from the clinical trial, and 3 patients were excluded because of loss of follow-up. Finally, a total of 139 patients in NS group were included in the analysis (Figure 1). In this study, 15 patients received rectal diclofenac to prevent post-ERCP pancreatitis. Among them, 9 patients (6.4%) were in the PPI group and 6 patients (4.3%) in the NS group, with no difference in the rate of rectal diclofenac administration (Table 2). Other baseline characteristics of participants in PPI and NS groups were also listed in Table 2.

**Table 2:** Baseline characteristics of participants in PPI and normal saline groups.

	PPI group	NS group
	(*n* = 141)	(*n* = 139)
Sex, *n* (%)		
Female	66 (46.8%)	72 (51.8%)
Male	75 (53.2%)	67 (48.2%)
Age, mean ± SD，years	56.23 ± 13.72	55.99 ± 14.41
Comorbid illness, *n* (%)		
Hypertension	28 (19.9%)	31 (22.3%)
Diabetes	5 (3.5%)	9 (6.5%)
Coronary artery disease	3 (2.1%)	6 (4.3%)
Cerebrovascular disease	6 (4.3%)	6 (4.3%)
Surgical history, *n* (%)		
Cholecystectomy	45 (31.9%)	35 (25.2%)
Major Gastrectomy	1 (0.7%)	2 (1.4%)
Concurrent medications *, *n* (%)		
Aspirin/Clopidogrel	3 (2.1%)	2 (1.4%)
Other NSAIDs	0	1 (0.7%)
Anticoagulants	1 (0.7%)	1 (0.7%)
Rectal diclofenac	9 (6.4%)	6 (4.3%)
Smoking, *n* (%)	29 (20.6%)	30 (21.6%)
Drinking, *n* (%)	19 (13.5%)	22 (15.8%)
Symptoms, *n* (%)		
Abdominal pain or distension	128 (90.8%)	126 (90.6%)
Nausea or vomiting	78 (55.3%)	58 (41.7%)
Jaundice	43 (30.5%)	51 (36.7%)
Fever	29 (20.6%)	40 (28.8%)
Others	9 (6.4%)	6 (4.3%)
Preoperative diagnosis, *n* (%)		
Choledocholithiasis (with or without cholangitis)	128 (90.8%)	131 (94.2%)
Malignant biliary stenosis	8 (5.7%)	4 (2.9%)
Bile duct cancer	4 (50.0%)	1 (25.0%)
Gallbladder cancer	1 (12.5%)	0
Duodenal papillary cancer	0	1 (25.0%)
Pancreatic cancer	3 (37.5%)	2 (50.0%)
Others	0	0
Benign or indeterminate biliary stenosis	3 (2.1%)	1 (0.7%)
Others	2 (1.4%)	3 (2.2%)

* Those concurrent medications were stopped at least 7 days prior to ERCP.

### 3.2. Laboratory test results

The pre-ERCP laboratory test results of the two groups were compared. Prothrombin time (PT) in the two groups was within the normal range, although the PPI group had a shorter PT than the NS group (*p* = 0.043). There were no significant differences in other laboratory tests such as blood routine examination, liver and kidney function between the two groups both before and after ERCP (*p* > 0.05) (Table 3).

**Table 3:** Laboratory test results of participants between the two groups before and after ERCP.

	ERCP	PPI group	NS group	PPI vs. NS
				*p* value
WBC (10^9/L)	Before	6.47 ± 2.60	6.62 ± 3.14	0.707
	After	7.21 ± 2.61	7.14 ± 2.50	0.766
Hb (g/L)	Before	131.61 ± 17.11	129.47 ± 26.53	0.422
	After	124.62 ± 16.45	125.27 ± 17.23	0.747
PLT (10^9/L)	Before	221.23 ± 79.80	216.93 ± 71.78	0.640
	After	218.23 ± 75.30	218.93 ± 71.57	0.706
TBIL(μmol/L)	Before	60.25 ± 97.91	54.42 ± 62.32	0.554
	After	45.84 ± 65.66	48.30 ± 63.65	0.751
BUN (mmol/L)	Before	4.46 ± 1.63	4.73 ± 1.68	0.173
	After	4.38 ± 1.73	4.61 ± 2.18	0.220
Cr (μmol/L)	Before	64.88 ± 34.27	66.29 ± 19.73	0.674
	After	69.31 ± 43.95	67.49 ± 19.46	0.783
PT (s)	Before	11.31 ± 1.30	11.63 ± 1.33	0.043
	After	11.44 ± 1.38	11.57 ± 1.10	0.393
INR	Before	1.00 ± 0.09	1.01 ± 0.13	0.387
	After	1.01 ± 0.09	1.01 ± 0.12	0.873

WBC, white blood cell count; Hb, hemoglobin; PLT, platelets; TBIL, total bilirubin; PT, prothrombin time; INR, international normalized ratio; BUN, blood urea nitrogen; Cr, creatinine.

### 3.3. ERCP-related results

In the PPI group, 4 cases (2.8%) had immediate bleeding during the EST procedure, and 5 cases (3.6%) in the normal saline group, with no statistical difference between the two groups (*p* > 0.05). All the immediate bleeding was stopped after successful endoscopic treatment before the end of ERCP. There were no significant differences between the two groups in terms of stone numbers, maximum diameter of stones, stone extraction methods, size of sphincterotomy incision, precut sphincterotomy, biliary duct dilatation method, the type of stent and so on (*p* > 0.05) (Table 4).

**Table 4:** ERCP procedure.

	PPI group (*n* = 141)	NS group (*n* = 139)	PPI vs. NS
			*p* value
Number of stones			
Single stone	47	51	0.556
Multiple stones	61	63	0.728
Muddy stone	21	19	0.770
Maximum diameter of stones			
<10 mm	82	81	0.984
10–15 mm	35	40	0.455
≥15 mm	12	12	0.971
Stone extraction method			
Stone basket	122	123	0.619
Stone balloon	8	6	0.602
Mechanical lithotripsy	9	10	0.787
The size of incision of EST			0.283
Small incision	52	39	
Moderate incision	80	103	
Large incision	9	11	
Precut	7	8	0.769
Biliary duct dilatation method			
Bouginage	7	8	0.796
Balloon dilation	50	50	0.929
ERBD			
Plastic stent	48	44	0.671
Covered self-expandable metal stents	2	3	0.683
Uncovered self-expandable metal stents	0	1	0.496
ENBD	36	43	0.315
Periampullary diverticulum			
Parapapillary diverticulum	45	40	0.568
Papilla in diverticulum	5	4	1.000
Post-EST immediate bleeding	4	5	0.749
SpyGlass+ SpyBite biopsy	0	1	0.496

EST, endoscopic sphincterotomy; ERBD, Endoscopic retrograde biliary drainage; ENBD, Endoscopic nose biliary drainage.

### 3.4. Post-EST delayed bleeding

Among all 280 patients eventually enrolled, a total of 6 patients had post-EST delayed bleeding, with the incidence of 2.14%, and the median occurrence time of delayed bleeding was 2.5 days after EST. Among them, 3 cases of delayed bleeding (2.12%, 3/141) were from the PPI group, of which one case had mild bleeding, manifested as melena within 24 h after ERCP, and the hemoglobin decreased by more than 3 g/dL compared with the pre-ERCP period, with no indication for blood transfusion. Another 2 cases had moderate bleeding, of which one patient vomited blood within 72 h after ERCP, and the other patient had melena on the 5th day after ERCP. Neither of them had the indication for blood transfusion. Another 3 cases of delayed bleeding (2.16%, 3/139) were from the NS group, of which 2 cases were mild, and one case was moderate, all of which were manifested as melena within 72 h after ERCP. All the patients with post-EST delayed bleeding had successful endoscopic treatment (Table 5), and there was no significant difference in the incidence and severity of post-EST delayed bleeding between the two groups (*p* = 1.000). All the bleeding were stopped after endoscopic treatment, and no patients underwent vascular embolization or surgical treatment, and no deaths occurred. We followed up all patients in this study by telephone or outpatient visit at the 1st week, 2nd week and 1st month after ERCP, and no patient presented with upper gastrointestinal bleeding such as hematemesis or melena.

**Table 5:** Post-EST delayed bleeding.

Patient	Group	Time of bleeding	Symptoms	Severity grading	Blood transfusions	Treatments
I	PPI	3rd day after EST	Hematemesis	Moderate	No	Ice saline rinsing + norepinephrine spray + argon plasma coagulation
II	NS	3rd day after EST	Melena	Moderate	Yes	Argon plasma coagulation
III	NS	1st day after EST	Melena	Mild	No	Titanium clip closure + argon plasma coagulation
IV	PPI	1st day after EST	Melena	Mild	No	Ice saline rinsing + titanium clip closure
V	NS	2nd day after EST	Melena	Mild	No	Argon plasma coagulation
VI	PPI	5th day after EST	Melena	Moderate	No	Argon plasma coagulation

### 3.5. Other post-ERCP adverse events

Among the 280 patients, the total incidence of post-ERCP adverse events rate was 11.79% (33/280), with no statistical difference between the PPI group (12.06%, 17/141) and the NS group (11.51%, 16/139). Besides post-EST bleeding, there were 22 cases of PEP (1.43%), 4 cases of biliary infection (1.43%), and 1 case of gastrointestinal perforation (0.36%) occurred. There was no significant difference in the incidence of adverse events between the two groups. There were no deaths in either group (Table 6). In this study, 22 patients with PEP relieved within 72 h after treatment with fasting, rehydration, inhibition of pancreatic enzymes with somatostatin, etc. Four patients with biliary tract infection improved after corresponding treatment such as anti-infection and rehydration. One patient with type III gastrointestinal perforation recovered after conservative treatment such as fasting, gastrointestinal decompression and rehydration.

**Table 6:** Incidence of adverse events after ERCP.

	PPI group	NS group	*p* value	Sum
	(*n* = 141)	(*n* = 139)		
Delayed bleeding	3 (2.12%)	3 (2.16%)	1.000	6 (2.14%)
Others				
PEP	13 (9.22%)	9 (6.47%)	0.393	22 (7.86%)
Biliary tract infection	0	4 (2.88%)	0.059	4 (1.43%)
Perforation	1 (0.71%)	0	1.000	1 (0.36%)
Death	0	0	1.000	0
Total	17 (12.06%)	16 (11.51%)	0.700	33 (11.79%)

## 4. Discussion

With the development of ERCP technology, EST has been continuously improved since it was introduced in 1974. At present, bleeding is still one of the serious adverse events of EST that cannot be ignored. It is well known that proton pump inhibitor (PPI) plays a significant role in the prevention and treatment of upper gastrointestinal bleeding (15, 16). However, whether using PPI after ERCP has a preventive effect on post-EST delayed bleeding still lacks sufficient evidence. On the other hand, there is an increasing number of studies showed that long-term use of PPI might cause a series of adverse events, such as increasing the incidence of gastrointestinal tumors, promoting the occurrence and development of atrophic gastritis, and increasing the risk of intestinal infections (17–19). More importantly, a recent study has shown that PPI is a risk factor for the recurrence of choledocholithiasis in patients underwent EST, and the possible mechanism is that PPI alters the intestinal microenvironment so as to cause excessive duodenal bacterial proliferation (20). Meanwhile, patients underwent EST have increased biliary reflux from duodenum (20). As a result, long-term PPI after EST increases the bacterial proliferation in bile duct, which in turn promotes the recurrence of choledocholithiasis (20). Therefore, whether it is necessary to routinely use PPI after EST to prevent post-EST bleeding is an important issue. Our study demonstrated that using PPI after ERCP did not prevent delayed bleeding after EST.

In our study, the overall incidence of post-EST bleeding was 2.14% (6/280), of which 5 cases (83.3%) of bleeding occurred within 72 h after ERCP, the other one occurred on the 5th day after ERCP, and the median time of delayed bleeding was 2.5 days after EST, which was consistent with previous reports (5–7). Bleeding is one of the most common adverse events after EST. There were many related studies on post-EST bleeding, but few studies involved the delayed bleeding, and the incidence of post-EST delayed bleeding has not been clarified, with large variation of results in previous reports (21–23). The variation of results might be due to the differences in patients’ characteristics, enrollment criteria, definition of delayed bleeding, and the operating skills of endoscopists. In our study, the patients’ baseline data showed that there was no significant difference between the two groups in the above-mentioned aspects, eliminating the interference of subjective factor such as endoscopists and patients on the results. In addition, we formulated strict inclusion and exclusion criteria, excluding patients with severe liver and kidney dysfunction, coagulation dysfunction, and those who need to receive anticoagulation or antiplatelet therapy within 1 week before ERCP. H2 receptor antagonists, mucosal protective agents, and hemostatic drugs that may affect the results were not used. As a result, the influence of other diseases and drug factors on post-EST delayed bleeding was minimized. Therefore, our study evaluated the role of PPI in preventing post-EST delayed bleeding as objectively as possible.

Among the 6 patients with post-EST delayed bleeding in this study, there were 3 patients in the PPI group and 3 in the NS group, with incidence rates of 2.12 and 2.16%, respectively. Among them, 2 patients in the PPI group had moderate bleeding and 1 patient had mild bleeding, while 1 patient in the NS group had moderate bleeding and 2 patients had mild bleeding. One patient (16.7%) with post-EST delayed bleeding in this study required blood transfusion. The remaining bleeding patients did not meet the indications for blood transfusion. There was no significant difference in the incidence and severity of post-EST delayed bleeding between the two groups (*p* = 1.000), which indicated that PPI has no significant effect on preventing post-EST delayed bleeding. It is well known that the mechanism of PPI in the prevention and treatment of upper gastrointestinal bleeding is to inhibit the secretion of gastric acid and increasing the intragastric pH level so as to prevent the dissolution of blood clots, and promote platelet aggregation and plasma coagulation so that the hemostatic effect works (24). Studies have shown that when the pH value is lower than 6, platelet aggregation and blood coagulation become abnormal, and when the pH value falls below 4, fibrin clot dissolved (25–27). Therefore, when PPI is used to prevent upper gastrointestinal bleeding, the pH should be maintained above 4. However, the pH value of the descending part of the duodenum where the duodenal papilla is usually located is above 4 in the natural environment (28), which might explain why there was no difference in the effect of using PPI versus normal saline on the incidence of post-EST delayed bleeding in this study.

Compared with the study of Leung et al. (13), our study is different in the following aspects. First, in the study by Leung (13), patients were enrolled before ERCP, and the patients who assigned to the experimental group were given PPI intravenously 4 h before ERCP to investigate the preventive effect of PPI on immediate bleeding and delayed bleeding after EST. However, previous studies have shown that immediate bleeding after EST is easy to find under endoscopy and can be dealt with in a timely manner, which has no clinical significance (2–4). Therefore, our study randomly assigned patients after ERCP, and focused on the preventive effect of PPI on post-EST delayed bleeding with the use of PPI or NS after ERCP immediately. Although previous studies have shown that immediate bleeding in EST is one of the risk factors for delayed bleeding and may increase the incidence of post-EST delayed bleeding (21, 29, 30), our study showed no statistical difference in the incidence of immediate bleeding between the PPI group and NS group (*p* > 0.05). All the immediate bleeding were also completely stopped during ERCP. Therefore, the potential contribution of post-EST immediate bleeding on delayed bleeding was equally distributed between the two groups. Second, in the research of Leung (13), the patients in the control group were only given standard ERCP care before and after ERCP, without a placebo control. However, in our study an equal number of NS (placebo control) was given intravenously at the same time in the control group, minimizing the possible bias of patients’ subjective factors. In addition, in Leung’s study (13), the second endoscopic examination was only performed in some patients with delayed post-EST bleeding, while the etiology of bleeding in other patients was not clear. In our study, all patients with suspected post-EST bleeding underwent second endoscopy to confirm the bleeding locus, and at the same time, the appropriate endoscopic treatment was performed to stop the bleeding, so that the interference by other bleeding reasons was excluded. However, this study has some limitations. First, we gave esomeprazole (Nexium) tablet to patients in the PPI group after 2 days, but did not give placebo tablet to patients in the NS group at the same time, which might be a source of possible performance bias of the methodology. Second, our study was a single-center study, so the sample size was relatively small and further evidence is needed in future multicenter studies.

In conclusion, this study shows that prophylactic use of PPI after EST does not reduce the incidence and severity of post-EST delayed bleeding. Therefore, PPI is not required for patients who underwent EST to prevent delayed bleeding, which can reduce unnecessary medication use in patients and reduce hospitalization costs, as well as avoid the abuse of PPIs, which leads to an increased incidence of related adverse effects.

## Data availability statement

The raw data supporting the conclusions of this article will be made available by the authors, without undue reservation.

## Ethics statement

The studies involving human participants were reviewed and approved by the Ethics Committee of the First Affiliated Hospital of Nanchang University. The patients/participants provided their written informed consent to participate in this study.

## Author contributions

ZYu, JHe, and RC contributed to conception and design of the study, data collection, and formal analysis, and wrote the first draft of the manuscript. ZYa and BL performed data collection and formal analysis, and wrote sections of the manuscript. JHo and YC performed project administration, data collection, and formal analysis. LZ contributed to conception and design of the study, funding acquisition, project administration, writing – review and editing. All authors contributed to the article and approved the submitted version.

## Funding

This study was supported by National Natural Science Foundation of China (No. 82160694), the project of the Jiangxi Department of Science and Technology (No. 20202BBGL73109), the project of the Health and Family Planning Commission of Jiangxi Province (No. 202310285), and Jiangxi Postgraduate Innovation Special Fund Project (YC2021-S192).

## Conflict of interest

The authors declare that the research was conducted in the absence of any commercial or financial relationships that could be construed as a potential conflict of interest.

## Publisher’s note

All claims expressed in this article are solely those of the authors and do not necessarily represent those of their affiliated organizations, or those of the publisher, the editors and the reviewers. Any product that may be evaluated in this article, or claim that may be made by its manufacturer, is not guaranteed or endorsed by the publisher.
